# Comparison of various anthropometric indices in predicting abdominal obesity in Chinese children: a cross-sectional study

**DOI:** 10.1186/s12887-019-1501-z

**Published:** 2019-04-24

**Authors:** Gengdong Chen, Huanchang Yan, Yuting Hao, Shiksha Shrestha, Jue Wang, Yan Li, Yuanhuan Wei, Jialiang Pan, Zheqing Zhang

**Affiliations:** 1 Foshan Institute of Fetal Medicine, Department of Obstetrics, Southern Medical University Affiliated Maternal & Child Health Hospital of Foshan, Foshan, 528000 Guangdong China; 2 Department of Nutrition and Food Hygiene, Guangdong Provincial Key Laboratory of Tropical Disease Research, School of Public Health, Southern Medical University, Guangzhou, 510515 China; 3 Department of Hygiene Detection Center, Guangdong Provincial Key Laboratory of Tropical Disease Research, School of Public Health, Southern Medical University, Guangzhou, 510515 China

**Keywords:** Abdominal obesity, Fat percentage, Anthropometric indicators, Children, Chinese

## Abstract

**Background:**

Former evidence regarding reference values of abdominal fat percentage (AFP) and optimal anthropometric indicators in predicting abdominal obesity measured by dual-energy X-ray absorptiometry (DXA) scan in Chinese children were scarce.

**Methods:**

A total of 452 Chinese children aged 6–9 years were included in this cross-sectional study. Abdominal fat and lean mass were measured by a DXA scan, and AFP were calculated. Anthropometric indicators including body mass index (BMI), chest circumference (CC), waist circumference (WC) and hip circumference (HC) were measured, waist-to-hip ratio (WHR), waist-to-height ratio (WHtR) was also calculated.

**Results:**

By defining abdominal obesity as those with an AFP ≥ 85th percentile, the cutoffs values are 24.80, 30.29, 31.58, 31.86% in boys, and 25.02, 30.32, 31.66, 31.79% in girls, for children aged 6, 7, 8, and 9 years old, respectively. All anthropometric indicators were independently and positively associated with AFP (*P* all < 0.01). In girls, BMI was found to be the optimal predictors of childhood abdominal obesity. The values of area under curves (AUCs) were significantly higher (*P* all < 0.05) than other anthropometric indicators, except for WHtR (AUCs value: 0.886). However, in boys, WHtR instead of BMI, provided the largest AUCs value (0.922) in predicting abdominal obesity, followed by BMI ((AUCs value: 0.913).

**Conclusion:**

This study provides reference values of AFP measured by DXA in Chinese children aged 6–9 years. BMI and WHtR tend to be the optimal anthropometric indicators in predicting abdominal obesity in Chinese girls and boys, respectively.

## Background

Childhood obesity has been increasing with an alarming rate globally and becoming one of the crucial medical issues threatening public health [1]. Extensive evidence indicates that obesity, especially abdominal obesity during childhood was associated with increased risks of metabolism syndrome [2], diabetes [3], and cardiovascular disease [4]. In 2015, 107.7 million children were obese worldwide; the overall prevalence was 5.0% [5]. While in China, the prevalence had been dramatically increased for overweight and obesity (from 5.0% to 19.2% during 1985 to 2010) [6], and especially for abdominal obesity (from 4.9% to 11.7% during 1993 to 2009) in children and adolescents aged < 18 years [7]. However, most of the previous studies used anthropometric indicators, like body mass index (BMI) or waist circumference (WC), for defining abdominal obesity, which might increase the possibility of misclassification since these indicators could not distinguish fat and lean mass precisely. Dual-energy X-ray absorptiometry (DXA) scans can provide direct and accurate measurement of the abdominal fat mass and distribution, and has been validated to be highly correlated with gold standards, like computed tomography [8], and magnetic resonance imaging [9]. However, until now, there is still lack of standardized cutoff value assessed by DXA to define abdominal obesity in Chinese children of early age.

Besides, most of the literature relies on BMI [10, 11], WC [12], waist-to-hip ratio (WHR) [13, 14] and waist-to-height ratio (WHtR) [10, 15, 16], to estimate the abdominal fat distribution. While few studies show relationship between other anthropometric parameters, like chest circumference (CC) and hip circumference (HC), and abdominal obesity [17, 18]. However, among a variety of anthropometric indicators, the most optimal one for predicting abdominal fat in Chinese children was still less clear.

Therefore, the objective of this study was to investigate the reference percentile curves for abdominal fat percentage (AFP) and to compare various anthropometric indicators (BMI, CC, WC, HC, WHR, and WHtR) in predicting abdominal obesity among children aged 6–9 years in China.

## Methods

### Study population

**Fig. 1:**
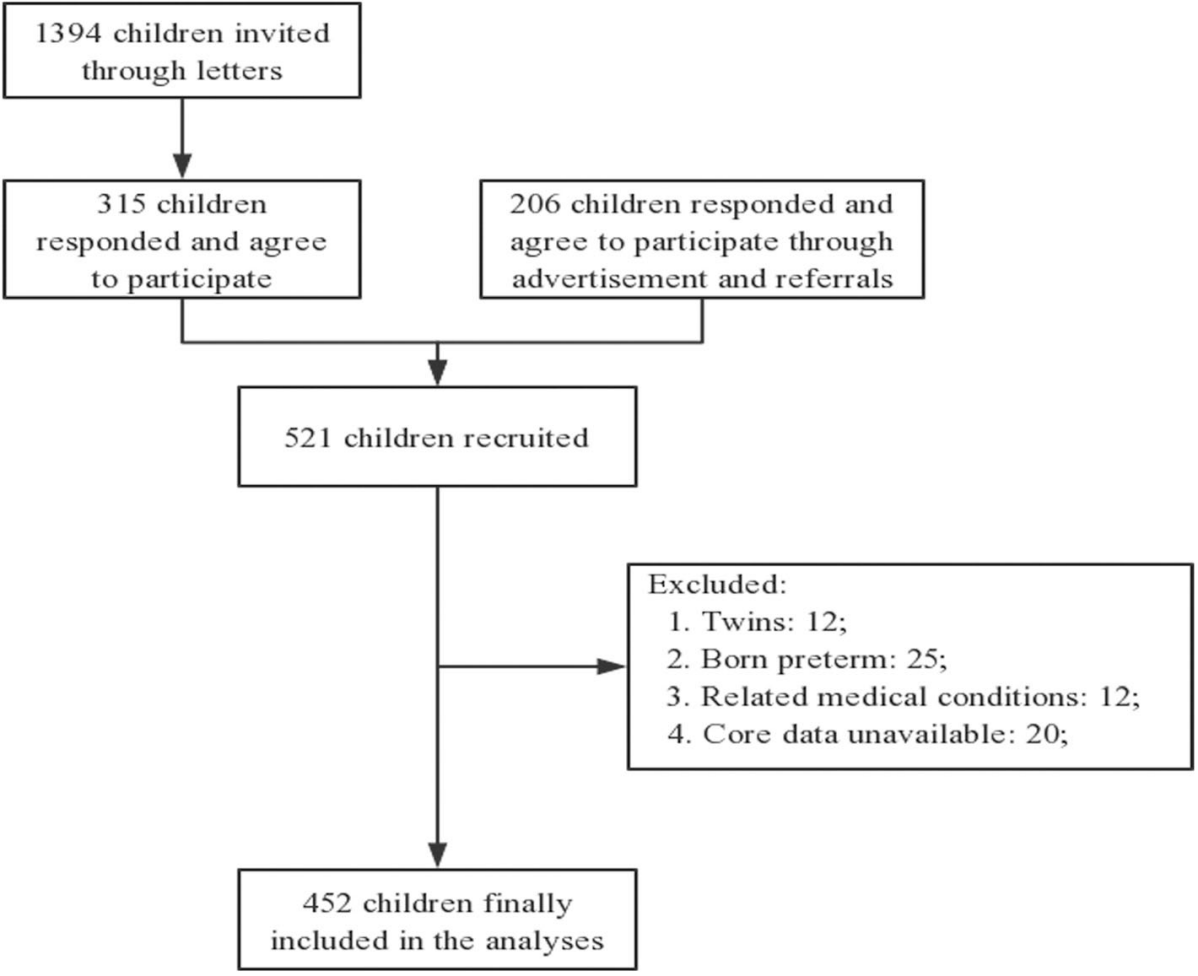
Flow chart of study participants

This cross-sectional study included 452-singleton birth children (255 boys and 197 girls) aged 6–9 years, who were recruited in urban Guangzhou, China, during December 2015 and March 2017. Two different ways were taken for the recruitment. One was by sending invitation letters with detailed criteria of inclusion and exclusion to several primary schools. 315 from a total of 1394 children responded and agreed to participate in the study. Another 206 children were enrolled through advertisements and referrals, bringing the total responding number to enroll to 521. We restricted the study to healthy, full-term singleton children aged 6–9 years, and subjects with the following criteria were excluded: twins (12); born pretermly (25); exposure to related medical conditions (12) that might have interfered with growth, including digestive tract disease, kidney stones or nephritis, thyrotoxicosis, hepatitis, anaphylactoid purpura, metabolic bone disease; Core data unavailable (20); Therefore, a total of 452 children aged 6–9 years were included in the final analyses (Fig. 1). All subjects were invited for physical examination.

### Anthropometry

Height and weight were measured with subjects in light clothing and shoes-off in standing position using a standard stadiometer and a Tanita MC-780A (Tanita Corporation, Tokyo, Japan) and accurate to 0.1 cm or kg. CC, WC, and HC were measured using inelastic tape around the same anatomical sites. Height, CC, WC, HC were measured to the nearest 0.1 cm and weight to the nearest 0.1 kg. All these measurements were operated twice, or thrice if differences larger than 2 cm was found, and the averages were calculated. BMI was calculated as weight (kg)/height square (m^2^). WHR was calculated as WC (cm)/HC (cm). WHtR was calculated as WC (cm)/height (cm).

### DXA scans

Abdominal fat and lean mass were measured with a whole-body DXA scanner (Discovery W; Hologic Inc., Waltham, MA, USA), and analyzed by the same experienced technician. Subjects wore only light clothing without metal or objects with high density, and hold the standard posture with the guide of technician during the scan. For quality control, a spine phantom was used for daily correction before formal scans. The coefficient of variation between two consecutive measurements with repositioning among 35 random selected children in the same day was 2.54% for abdominal fat mass.

### Statistical analysis

The data from boys and girls were analyzed separately and presented as Mean ± standard deviation (SD) for the continuous variables and as frequencies and percentages for the categorical variables. Student’s t test was used to ascertain the significance of the difference in the continuous variables between boys and girls.

We calculated age- and sex-specific Z-scores and established age- and sex-specific reference values for AFP using LMSChartmaker 2.54 (Medical Research Council, London, UK). AF*P* values of each child were compared with corresponding, newly developed age- and sex-specific reference values to estimate Z-scores and percentiles. Multivariate linear regression models were operated to examine the agreement between AFP and Z-scores for BMI, CC, WC, HC, WHR and WHtR after adjusting for age (six pairs), stratified by sex. Area under the receiver-operating characteristic (ROC) curves were drawn with the help of MedCalc® version 11.4.2.0 for Windows for estimating the screening of abdominal obesity (AFP ≥ 85th percentile) by using different anthropometric measures, stratified by sex. Values of area under curve (AUC) were estimated. Other analyses were operated using IBM SPSS 20.0 (Chicago, IL, USA) and a two-side *P* value of < 0.05 was considered statistically significant.

## Results

### Characteristics of subjects

The characteristics of subjects are shown in Table 1. The study included 255 (56.4%) boys and 197 (43.6%) girls. The mean ages were 7.97 ± 0.91 years for boys and 8.06 ± 0.95 years for girls. The prevalence of abdominal obesity is 20.4% in boys and 16.8% in girls. Compared with girls, boys tend to have higher values of weight, BMI, CC, WC, WHR and WHtR (*P* all < 0.05). No differences were found in average age, height, HC and AFP between boys and girls (*P* > 0.05).

**Table 1:** Selected characteristics of the study population

Variables	Boys			Girls			Total
	Obesity (*n* = 52)	Non-obesity (*n* = 203)	Total (*n* = 255)	Obesity (*n* = 33)	Non-obesity (*n* = 164)	Total (*n* = 197)	*P-*value ^a^
Age (years)	8.17 ± 1.03	7.92 ± 0.88	7.97 ± 0.91	7.88 ± 0.97	8.10 ± 0.95	8.06 ± 0.96	0.285
Height (m)	1.34 ± 0.09^***^	1.28 ± 0.08	1.29 ± 0.08	1.30 ± 0.08	1.28 ± 0.08	1.28 ± 0.08	0.679
Weight (kg)	37.1 ± 10.4^***^	24.8 ± 4.58	27.3 ± 7.93	31.4 ± 6.47^***^	24.1 ± 4.43	25.3 ± 5.53	0.002
BMI (kg/m^2^)	20.4 ± 3.77^***^	15.1 ± 1.66	16.2 ± 3.09	18.3 ± 2.18^***^	14.6 ± 1.44	15.2 ± 2.10	< 0.001
CC (cm)	70.5 ± 9.59^***^	59.1 ± 4.05	61.4 ± 7.26	64.8 ± 5.85^***^	57.5 ± 3.94	58.7 ± 5.10	< 0.001
WC (cm)	68.8 ± 10.5^***^	54.4 ± 4.58	57.4 ± 8.52	61.5 ± 6.85^***^	52.8 ± 4.16	54.2 ± 5.71	< 0.001
HC (cm)	77.1 ± 9.18^***^	64.2 ± 5.36	66.8 ± 8.17	72.8 ± 6.30^***^	64.2 ± 5.16	65.6 ± 6.25	0.07
WHR	0.89 ± 0.05^***^	0.85 ± 0.04	0.86 ± 0.04	0.84 ± 0.05^*^	0.82 ± 0.04	0.83 ± 0.05	< 0.001
WHtR	0.51 ± 0.06^***^	0.42 ± 0.03	0.44 ± 0.05	0.47 ± 0.04^***^	0.41 ± 0.03	0.42 ± 0.04	< 0.001
AFP (%)	35.5 ± 5.07^***^	20.7 ± 4.24	23.7 ± 7.43	35.3 ± 5.15^***^	22.8 ± 4.39	24.9 ± 6.48	0.08

*BMI* Body Mass Index, *CC* Chest Circumference, *HC* Hip Circumference, *WC* Waist Circumference, *WHR* Waist-to-Hip Ratio, *WHtR* Waist-to-Height Ratio, *AFP* Abdominal fat percentage

^a^test for differences between boys and girls. ^*^: *P* < 0.05; ^**^: *P* < 0.01; ^***^: *P* < 0.001 compared with the non-obesity groups

### AFP percentile curves

**Fig. 2:**
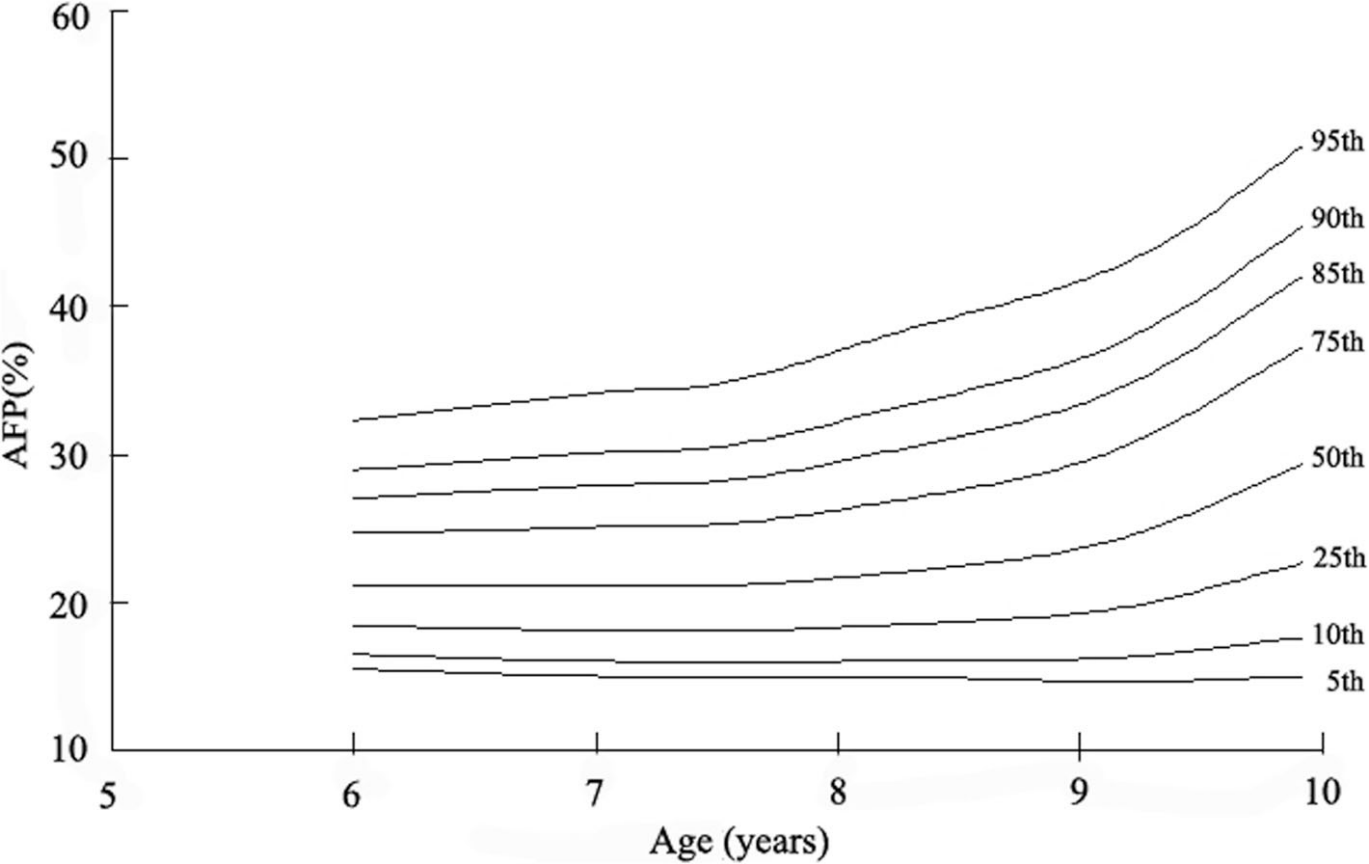
Reference percentile curves of abdominal fat percentage for boys

**Fig. 3:**
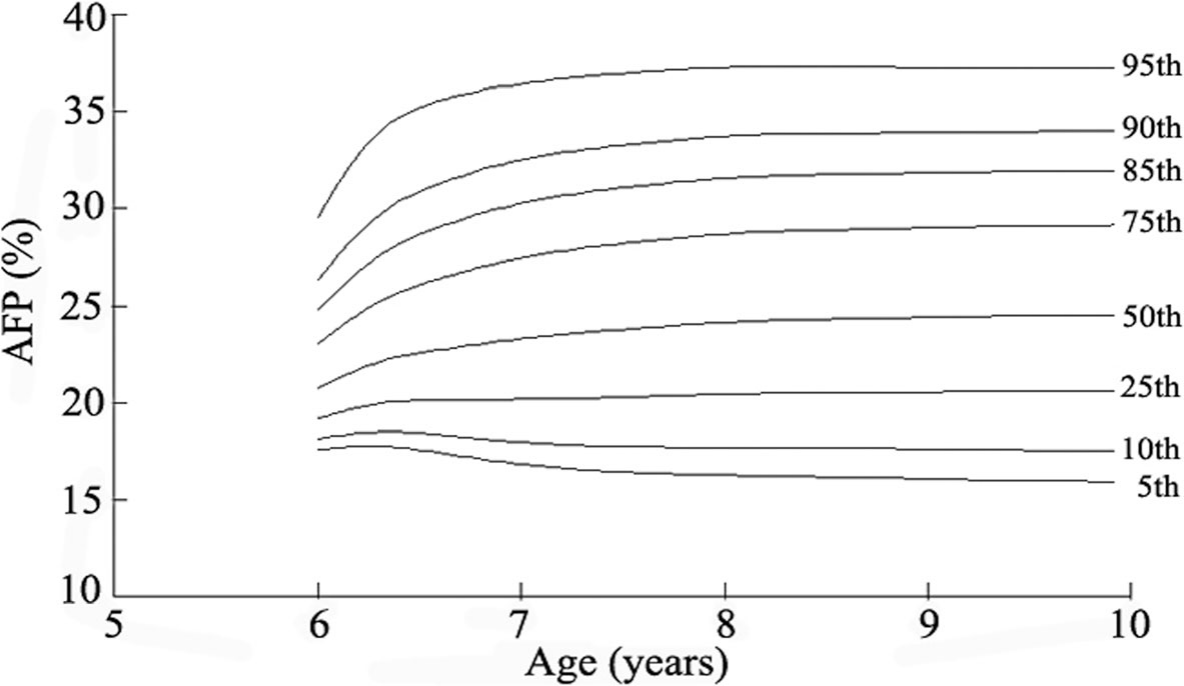
Reference percentile curve of abdominal fat percentage for girls

The reference percentile curves derived for AFP for boys and girls by age are illustrated in Figs. 2 and 3. Growth curves providing the 5th, 10th, 25th, 50th, 75th, 85th, 90th, 95th centiles for AFP in boys and girls and equivalent percentile values are given in Table 2. The AFP of participants used to classify as abdominal obesity (AFP ≥ 85th percentile). The cutoff values of AFP in defining abdominal obesity among children aged 6, 7, 8, 9 years old are 24.80, 30.29, 31.58, and 31.86%, respectively in boys and 25.02, 30.32, 31.66, and 31.79%, respectively in girls.

**Table 2:** Smoothed percentiles for abdominal fat percentage among boys and girls aged 6–9 years

Age (years)	Percentile for boys (%)								Percentile for girls (%)							
	5th	10th	25th	50th	75th	85th	90th	95th	5th	10th	25th	50th	75th	85th	90th	95th
6	17.57	18.12	19.20	20.78	23.06	24.80	26.34	29.57	17.86	18.38	19.42	20.96	23.23	25.02	26.67	30.38
7	16.83	17.95	20.17	23.31	27.46	30.29	32.53	36.46	16.61	17.78	20.09	23.31	27.50	30.32	32.51	36.30
8	16.27	17.69	20.44	24.13	28.69	31.58	33.75	37.30	16.35	17.75	20.45	24.13	28.72	31.66	33.88	37.56
9	16.07	17.61	20.55	24.41	29.02	31.86	33.94	37.29	16.17	17.70	20.60	24.42	28.98	31.79	33.85	37.17

### Relationships between age-adjusted anthropometric indicators and AFP

Regression coefficient (*β*) between age-adjusted anthropometric indicators and AFP were shown in Table 3. All anthropometric indicators were significantly and positively associated with AFP. BMI tend to provide the largest coefficients in girls but not in boys. Per one SD increase of relative anthropometric indicators, AFP increased by 3.173% to 6.632% in boys and 1.634% to 5.111% in girls.

**Table 3:** Relationships of age-adjusted physical indicators for assessing abdominal fat percentage in boys and girls

Variables	Boys			Girls		
	*β* ^a^ (%)	*β* ^b^ (%)	*P* value	*β* ^a^ (%)	*β* ^b^ (%)	*P* value
BMI	6.209	0.835	< 0.001	5.111	0.789	<0.001
CC	6.389	0.860	< 0.001	4.781	0.738	<0.001
WC	6.379	0.858	< 0.001	4.854	0.749	<0.001
HC	6.632	0.892	< 0.001	4.994	0.770	<0.001
WHR	3.173	0.427	< 0.001	1.634	0.252	0.001
WHtR	5.845	0.786	< 0.001	4.861	0.750	<0.001

Per one standard deviance increase of anthropometric indicators

*BMI* Body Mass Index, *CC* Chest Circumference, *HC* Hip Circumference, *WC* Waist Circumference, *WHR* Waist-to-Hip Ratio, *WHtR* Waist-to-Height Ratio

^a^: unstandardized regression coefficients . ^b^: standardized regression coefficients

### Performance of anthropometric measures

AUC was used to evaluate the performance of each anthropometric indicator for the screening of abdominal obesity (AFP ≥ 85th) by sex. As shown in Table 4, BMI and WHtR exhibited the largest AUC in both boys (AUC = 0.913 and 0.922) and girls (AUC = 0.925 and 0.886). For other indicators (CC, WC, HC, WHR), AUC values ranged from 0.744 to 0.898 in boys and from 0.605 to 0.869 in girls. Significant higher AUC were found for BMI compared to other indicators expect for WHtR in girls (*P* < 0.01), and CC and WHR, but not WC, HC, WHtR in boys. For both boys and girls, WHR performed were poorest in predicting abdominal obesity by providing least AUC values (0.744 in boys and 0.605 in girls), which were significantly lesser than those observed for BMI or WHtR (*P* < 0.001).

**Table 4:** Comparison of the Receivers Operator Characteristic curves for various anthropometric indices in predicting abdominal obesity

Variables		AUC	*95% CI*	Sensitivity (%)	Specificity (%)	*P* value ^a^	*P* value ^b^
Boys	BMI	0.913	0.872, 0.945	73.1	95.1	–	0.542
	CC	0.870	0.822, 0.908	76.9	85.2	0.017	0.041
	WC	0.898	0.854, 0.932	78.9	87.2	0.307	0.247
	HC	0.882	0.836, 0.919	69.2	92.6	0.057	0.114
	WHR	0.744	0.686, 0.796	59.6	76.4	< 0.001	< 0.001
	WHtR	0.922	0.882, 0.952	80.8	88.7	0.542	–
Girls	BMI	0.925	0.879, 0.958	90.9	87.6	–	0.217
	CC	0.852	0.794, 0.898	72.7	86.0	0.007	0.472
	WC	0.863	0.807, 0.908	69.7	88.4	0.006	0.431
	HC	0.869	0.814, 0.913	87.9	76.2	0.006	0.712
	WHR	0.605	0.533, 0.674	36.4	86.6	< 0.001	< 0.001
	WHtR	0.886	0.833, 0.926	81.8	80.5	0.217	–

*BMI* Body Mass Index, *CC* Chest Circumference, *HC* Hip Circumference, *WC* Waist Circumference, *WHR* Waist-to-Hip Ratio, *WHtR* Waist-to-Height Ratio

^a:^Compared with BMI. ^b:^ Compared with WHtR

## Discussion

According to our knowledge, this is the first study to develop age- and gender-specific reference percentiles for AFP measured by DXA for Chinese children. Besides, we further found that BMI and WHtR, compared with other indicators, performed optimally in predicting abdominal obesity in Chinese girls and boys, respectively.

Former evidence had indicated that obesity; especially abdominal obesity in early childhood might increase the risk of later chronic diseases [4–7]. It is important to explore the reference values of the abdominal obesity measured by more precisely methods, like DXA. However, the corresponding reference values had not been established in Chinese children before. Using the available data, we filled the gap on this field. Besides, considering attenuated time and economic expenditure, it would be of great utility value to investigate the most optimal anthropometric indicators correlated with abdominal obesity measured by DXA, when applied in large epidemiology surveys.

In our study, we observed that BMI tend to be the optimal indicator of abdominal obesity in young Chinese children aged 6–9 years, especially in girls. In consistent with our results, several studies showed BMI was highly correlated to abdominal fat. Dencker et al. found strong correlation between BMI and abdominal fat mass in Swedish children (*r* = 0.93–0.95) [19]. Moreover, based on Japanese children population, BMI was also recommended as a screening tool to identify abdominal adiposity. The researchers suggested that the optimal cut-off values for BMI were 20 kg/m^2^ for boys (sensitivity: 100%, specificity: 90%) and 19 kg/m^2^ for girls (sensitivity: 100%, specificity: 90%) [10]. However, there are other studies that claim BMI might give less indication of fat distribution [6, 20, 21], and might be interfered by fat free mass [22]. Accordingly, few studies suggested that the measurement of BMI was needed in addition to WC [6] or WHtR [19]. Former evidence indicated WC [10, 23–26] and WHtR [10, 20] as good indicators in predicting abdominal obesity in children, however, BMI was more superior compared with WC in girls and not different with WHtR in predicting childhood abdominal obesity in our study. The divergent conclusions might be sources from the difference of population studied. Children in China and Japan tend to be with lower BMI or obesity degree than those from the western countries. Therefore, relative less fat is deposited at the abdomen, and then might attenuate the utility of WC and related indicators, especially in children. More studies were needed for better illustration of the problem.

WHR was found as a poor predictor of childhood abdominal obesity in our study, the results were consistent with several other studies [23, 25] . Taylor et al. showed that WHR was poorly associated with central adiposity [25]. The use of WHR to assess abdominal obesity in children might not be appropriate because this ratio is highly age dependent [27]. Our results together with former evidence, suggested that WHR might be of less value in predicting abdominal obesity in children.

One of the strengths of this study was that we provided the first reference data of AFP based on Chinese children aged 6–9 years. Additionally, by comparing several anthropometric indicators, we found that BMI and WHtR tended to perform optimally in predicting childhood abdominal obesity, which might provide more specific guidance for large epidemiology surveys focus on childhood obesity. There were also several limitations in our study. Firstly, due to the absence of standard cut-off for AFP in Chinese children, we used the 85% value as a cut-off to determine abdominal obesity. However, this cut-off value might be likely to differ in different populations. Secondly, with the cross-sectional design, we fail to investigate the best anthropometric indicators in predicting the dynamic trajectory of abdominal obesity in children. Thirdly, the study was based on a relatively small sample of children with a limited age range; more studies with large samples and wider age range were needed to reexamine our results. Lastly, the measurement of neck circumference and sexual development assessment were not performed in the study. Therefore, we could not perform further analyses on these fields, which were encouraged to be involved in further studies.

## Conclusions

We present the first reference data for AFP in Chinese children aged 6–9 years. Compared with other anthropometric indicators, BMI and WHtR tend to perform optimally in predicting childhood abdominal obesity.

## Abbreviations

AFP: Abdominal fat percentage
AUC: Area under curve
BMI: Body mass index
CC: Chest circumference
DXA: Dual-energy X-ray absorptiometry
HC: Hip circumference
SD: Standard deviation
WC: Waist circumference
WHR: Waist-to-hip ratio
WHtR: Waist-to-height ratio
